# Orbital Resizing

**DOI:** 10.5935/0004-2749.2023-1006

**Published:** 2023

**Authors:** Fernando Procianoy, Antonio Augusto Velasco e Cruz

**Affiliations:** 1 Hospital de Clínicas de Porto Alegre, Faculdade de Medicina, Universidade Federal do Rio Grande do Sul, Porto Alegre, RS, Brazil; 2 Hospital das Clínicas, Faculdade de Medicina de Ribeirão Preto, Universidade de São Paulo, Ribeirão Preto, SP, Brazil

**Figure 1.**
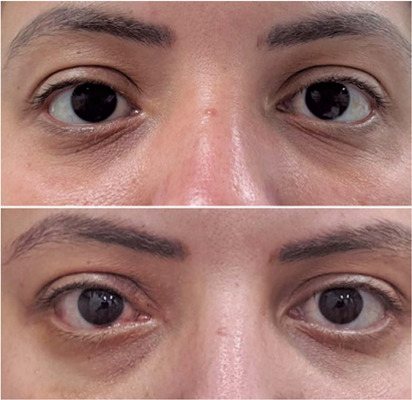
Cosmetic pre- and post-orbital resizing (ethmoidectomy) performed to correct residual right eye exophthalmos in a patient with a history of ethmoidal sinus mucocele drainage.

Orbital decompression is a recognized oculoplastic surgery procedure, initially used for treating compressive optic neuropathy and facial disfigurement in thyroid eye disease^(1,2,3,4,5)^. The advancement of techniques and integration of technology have resulted in safer, more efficient procedures. Currently, surgeons can choose to remove orbital fat and/or reshape several orbital bone walls in different ways and combinations to achieve desired outcomes. These advancements have ushered in a new era of orbital surgery, targeting exophthalmos reduction in patients with or without thyroid eye disease (Figure 1), even without compressive orbitopathy^(6,7)^.

Despite alterations in patient profiles and indications, surgeons persist in using the term “orbital decompression” to refer to this wide variety of diseases and surgical indications. In an era where monoclonal antibody treatments also address exophthalmos reduction, it might be time to rethink the naming of surgical procedures based on their main indications. The term “orbital decompression” seems incorrect when used for a procedure conducted in an orbit without any compression Symptoms such as optic neuropathy or vascular congestion.

Most of the orbital surgeries we perform today on patients with thyroid eye disease aim to reduce exophthalmos, without any actual orbital compression. For this patient profile, expectations and concerns differ from patients with sight-threatening diseases or significant functional issues such as diplopia. The differences in surgeon and patient expectations, procedure selection, and postoperative care require distinguishing between these two types of procedures-for patient comprehension, accurate documentation, and even for billing purposes.

Therefore, we suggest using the term “orbital resizing” for exophthalmos-reduction procedures performed on patients without actual orbital compression, at the discretion of each surgeon.
